# UVB Irradiation Induced Cell Damage and Early Onset of *Junbb* Expression in Zebrafish

**DOI:** 10.3390/ani10061096

**Published:** 2020-06-25

**Authors:** Rui-Yi Chen, Chun-Ju Lin, Sung-Tzu Liang, Omar Villalobos, Oliver B. Villaflores, Bao Lou, Yu-Heng Lai, Chung-Der Hsiao

**Affiliations:** 1 Key Lab of Mariculture and Enhancement of Zhejiang Province, Marine Fisheries Research Institute of Zhejiang, Zhoushan 316100, China; xx_cry@163.com; 2 Marine and Fishery Institute, Zhejiang Ocean University, Zhoushan 316100, China; 3 Department of Bioscience Technology, Chung Yuan Christian University, Chung-Li 32023, Taiwan; cdhsiao@yahoo.com.tw (C.-J.L.); stliang3@gmail.com (S.-T.L.); 4 Department of Pharmacy, Faculty of Pharmacy, University of Santo Tomas, Manila 1015, Philippines; oavillalobos@ust.edu.ph; 5 Department of Biochemistry, Faculty of Pharmacy, University of Santo Tomas, Manila 1013, Philippines; obvillaflores@ust.edu.ph; 6 Institute of Hydrobiology, Zhejiang Academy of Agricultural Sciences, Shiqiao Road 198, Hangzhou 310021, China; 7 Department of Chemistry, Chinese Culture University, Taipei 11114, Taiwan; 8 Department of Chemistry, Chung Yuan Christian University, Chung-Li 32023, Taiwan; 9 Center for Nanotechnology, Chung Yuan Christian University, Chung-Li 32023, Taiwan

**Keywords:** UVB, zebrafish, skin, biomarker, microarray

## Abstract

**Simple Summary:**

Zebrafish is a good in vivo model to study how skin responds to Ultraviolet B (UVB) irradiation at the cellular, molecular, and whole organism levels. Previous studies showed that zebrafish embryo fin undergoes extensive shrinkage after exposure to UVB irradiation, and this phenotypic change can be assessed using antioxidant drugs. To provide more detailed chronological changes for zebrafish embryos after receiving UVB irradiation, sequential alterations of zebrafish embryos at morphological (fin), cellular (cell death, oxidative stress, immune-response, and marker gene expression) and molecular (microarray screen and real-time RT-PCR assay) levels were examined in this study. The results showed that *junbb* gene expression was activated as early as 3 h post-UVB irradiation, followed by significant elevation of apoptosis around 9 h post-UVB irradiation, neutrophil migration to the wound area approximately 14 h post-UVB irradiation, and activation of *mmp* gene expression at around 24 h post-UVB irradiation. These chronological cellular and molecular responses after UVB irradiation in zebrafish provide a basic and fundamental foundation for future line construction and UVB-associated gene validation.

**Abstract:**

Ultraviolet B (UVB) radiation has drawn more attention over these past few decades since it causes severe DNA damage and induces inflammatory response. Serial gene profiling and high throughput data in UVB-associated phenomenon in human cultured cells or full rack of human skin have been investigated. However, results using different tissue models lead to ambiguity in UVB-induced pathways. In order to systematically understand the UVB-associated reactions, the zebrafish model was used, and whole organism gene profiling was performed to identify a novel biomarker which can be used to generate a new mechanistic approach for further screening on a UVB-related system biology. In this study, detailed morphological assays were performed to address biological response after receiving UVB irradiation at morphological, cellular, and molecular levels. Microarray screening and whole genome profiling revealed that there is an early onset expression of *junbb* in zebrafish embryos after UVB irradiation. Also, the identified novel biomarker *junbb* is more sensitive to UVB response than *mmps* which have been used in mouse models. Moreover, cellular and molecular response chronology after UVB irradiation in zebrafish provide a solid and fundamental mechanism for use in a UV radiation-associated study in the future.

## 1. Introduction

Ultraviolet radiation, categorized by wavelengths into UVC (200–280 nm), UVB (280–320 nm), and UVA (320–400 nm), has the ability to ionize molecules and induces a series of chemical reactions [1]. Generally, about 95% of the UV light reaching the ground is UVA. Both UVC and UVB are mostly filtered and absorbed by Earth’s atmosphere, such as the ozone, oxygen, and water, during light transmission. Due to the fact of ozone layer depletion, an increase in exposure of the Earth’s surface to UVB and UVC radiation has been observed [2,3].

A small amount of UV radiation is beneficial for human metabolism [4]. It is also applied to medical treatments such as jaundice in newborn infants [5] and rickets in geriatrics [6]. However, overexposure to solar UV radiation causes acute and chronic health issues that may be irreversible. Excess UVA causes “photo aging”, resulting in lackluster skin with wrinkles [7]. In addition, UVA induces synthesis of matrix metalloproteinase (MMP) which decomposes collagen fibrils and inhibits procollagen synthesis, leading to disorganized dermal skin tissue structure [8]. Moreover, cells produce reactive oxygen species (ROS) after prolonged exposure to UVA which causes DNA damage, unbalances the immune system, and, finally, leads to skin cancer [9]. It has been proved that UVA is associated with malignant melanoma which is one of the fastest spreading cancers in humans [10]. On the other hand, although UVB may only play a role in non-melanoma skin cancer, which is associated with cytochromes P450 [11], its high energy causes serious DNA damage by formation of photoreactive pyrimidine dimmers, such as cyclobutane-pyrimidine dimers (CPDs) and pyrimidine-pyrimidone (6–4) photoproducts (6–4 PP), leading to mutagenesis and carcinogenesis on skin tissues [12,13]. These two major photoproducts damage DNA by making lesion, causing distortion of DNA structure and interrupting DNA replication and transcription [14].

The immune system is also affected by long-term exposure to UV radiation. The UV-induced homeostasis and immune response were crucial for the neuroendocrine network through daily skin exposure [15,16]. It is suggested that UVB induces serious inflammatory reaction via tumor necrosis factor-α (TNF-α) and inflammatory factors, IL-6, and IL-10 in the cyclic adenosine monophosphate (cAMP)/protein kinase A pathway [17]. Also, UV-induced neutrophilic inflammatory response, including the release of high mobility group box 1 (HMGB1) and Toll-like receptor 4 (TLR4), promoted angiogenesis and metastasis in melanoma cells [18]. A well-established zebrafish model for UV-induced inflammatory response has been published indicating that UV exposure causes inflammation and mortality during embryogenesis [19].

In a previous study, whole gene profiling on the full thickness human skin after UVB irradiation has been reported and unique target genes were identified. For example, *CCL2*, *CXCL1*, and *IL-8* were significantly upregulated, and which were associated with multiple innate and early adaptive immune pathways [20]. Also, UVB-dependent activation on cutaneous *HSD11B1* expression has been demonstrated as a key regulator of cortisol activity in epidermal homeostasis [21]. However, when using human keratinocytes as a UVB-induced responsive model, different cellular biomarkers were identified [22]. Generally, a multi-layer of epidermis and the underlying dermis with collagenous stroma in between were fully developed in adult zebrafish that showed a similar structure with humans [23]. In addition, the skin development of zebrafish has been reported as under sonic hedgehog control [24] which is involved in many types of carcinogenesis in human, including melanoma [25,26]. This may suggest many advantages of studying human cutaneous disease with zebrafish instead of mice [23]. With the breakthrough findings on the UVB-induced biomarker in our study, the zebrafish model has the potential to be a platform in the future for screening drugs that diminish UVB-caused damage such as various mutagenic DNA lesions [14].

## 2. Materials and Methods

### 2.1. Zebrafish Keeping and Ethics

All experimental protocols and procedures involving zebrafish were approved by the Committee for Animal Experimentation of the Chung Yuan Christian University (Number: CYCU104024, issue date 21 December 2015). All experiments were performed in accordance with the guidelines for laboratory animals. Wild-type AB strain zebrafish were maintained in a recirculating aquatic system at 28.5 °C. Circulating water in the aquarium was filtered by reverse osmosis (pH 7.0–7.5). The zebrafish were fed twice a day with lab-grown brine shrimp.

### 2.2. Zebrafish UVB Irradiation

The zebrafish embryos (3 days post-fertilization, dpf) were placed on a glass slide with a cover slip, and excess water was carefully removed by pipetting to a level of around 50–100 μL. Next, the embryos were placed into a UV crosslinker (Spectroline, XLE-1000B, UV spectrum 280–380 nm, WL = 312 nm) and exposed to UVB at different dosages from 0 to 1000 J/m^2^. After irradiation, embryos were placed back into fish water and incubated at 28.5 °C for further experiment.

### 2.3. Histology

Plastic section of the caudal fin of zebrafish embryo was subjected for histological analysis. Zebrafish larvae aged 4 dpf were fixed overnight in 4% paraformaldehyde at 4 °C and then dehydrated overnight in 100% methanol at −20 °C. After complete dehydration, samples were infiltrated and embedded in Technovit 7100 resin (Heraeus Kulzer, Hanau, Germany). Samples were sectioned at 2 μm intervals and stained with a hematoxylin and eosin staining kit (Merck, Fort Kenalworth, NJ, USA).

### 2.4. Acridine Orange Staining

The UVB irradiated embryos were transferred into a 24 well plate and then incubated at dark in 2 μg/mL Acridine Orange (A3568, Invitrogen, Carlsbad, CA, USA) solution for 15 min. Washing of embryos was done repeatedly to remove excess dyes. 

### 2.5. ROS Detection

The UVB irradiated embryos were transferred into a 24 well plate and then incubated in 40 μM DCF-DA (2’,7’-dichlorofluorescin diacetate) (D399, Invitrogen) staining solution in the dark for 30 min. Excess dyes were washed from embryos three times. The DCF-DA-positive staining area was measured by ImageJ software.

### 2.6. Whole-Mount Immunostaining

Zebrafish embryos aged at specific developmental stages were fixed in 4% paraformaldehyde/PBS for 12 h at 4 °C. After successive washing with PBST, embryos were transferred to 100% methanol and stored at −20 °C for 2 h and were further subjected to rehydration with PBST. After blocking with 3% BSA/PBST at room temperature for 60 min, embryos were incubated at 4 °C overnight with 1:200 diluted primary antibodies as follows: polyclonal rabbit anti-p53 antibody (GTX102965, GeneTex, Irvine, CA, USA), polyclonal rabbit anti-Ku80 antibody (GTX109935, GeneTex), polyclonal rabbit anti-Rad51 (GTX100469, GeneTex). After incubation, embryos were washed with PBST for 10 min and were further incubated with 1:500 diluted Alexa Fluor 488-conjugated secondary antibodies (Invitrogen) for fluorescent signals. To visualize the nuclear position, some embryos were counter-stained with Hoechst 33342 (Invitrogen).

### 2.7. Quantitative Real-Time-PCR

Thirty embryos at specific developmental stages were collected and homogenized in RNAzol RT (RN190, MRC, Inc., Houston, TX, USA) with a Bullet Blender tissue lyser (Next Advance, Inc., Troy, NY, USA) to isolate the total RNA using the manufacturer’s protocol. Total RNA concentration was determined by spectrophotometry, and the RNA quality was checked by electrophoresis in denatured gels. For qRT-PCR, 1 μg of total RNA was reverse-transcribed with RevertAid first cDNA synthesis kit (K1622, Fermentas, Vilnius, Lithuania) and then PCR was performed with SYBR green dye according to the manufacturer’s instructions. The primer sequences used to perform qRT-PCR and its amplicon size are listed in Table S1. The *β-actin* gene was used as a housekeeping gene for relative gene expression normalization, since this gene displays stable expression in zebrafish embryos receiving with or without UVB irradiation [27,28].

### 2.8. Image Acquisition, Quantification, and Statistics

Representative fluorescent images were acquired using an upright microscope (BX51, Olympus, Tokyo, Japan) equipped with a digital camera (DP72, Olympus) or a dissecting microscope (SMZ1500, Nikon, Tokyo, Japan) equipped with a cool CCD (Evolution VF, Denver, CO, USA). For quantifying the relative caudal fin size, the original images captured at the caudal fin position were processed using Photoshop CS3 software to select a region of interest (ROI) at 150 μm × 450 μm dimensions. The total cell number in this ROI was calculated using ImageJ software and statistically compared using either *t*-test or one-way ANOVA.

### 2.9. Microarray Analysis

The GeneChip Zebrafish Genome Array was purchased from Affymetrix and contained 14,900 transcripts. The details of oligonucleotide description can be obtained from Affymetrix website. Thirty non-irradiated and UVB irradiated 4 dpf-old embryos were collected and subjected for the extraction of total RNA using Trizol reagent (Invitrogen). The probe synthesis and array hybridization were operated by Microarray & Gene Expression Analysis Core Facility at National Yang-Ming University according to the standard protocol. The microarray data were submitted to NCBI Gene Expression Omnibus.

### 2.10. Statistical Analysis

All statistics are expressed as means ± SD and tested by student *t*-test or one-way ANOVA. Values of *p* < 0.005 were considered statistically significant. The experiments were repeated at least three times independently.

## 3. Results

### 3.1. Optimization of UVB-Induced Zebrafish Model

**Figure 1:**
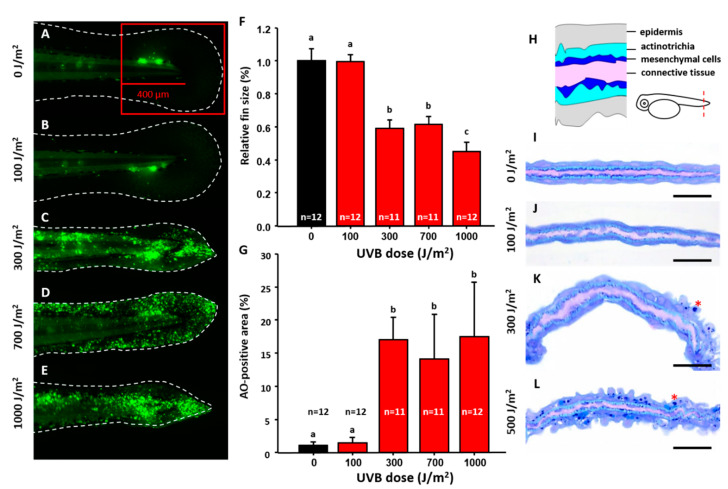
Optimization of UVB irradiation dosage to induce caudal fin damage in zebrafish. Zebrafish embryos aged 3 dpf were exposed to UVB irradiation at different doses of 0 (**A**), 100 (**B**), 300 (**C**), 700 (**D**), and 1000 (**E**) J/m^2^. Twenty-four hours post irradiation, embryos were stained with acridine orange (AO) to detect apoptosis and the relative size of caudal fin was calculated and compared. (**F**) Quantitative measurement of caudal fin area under different doses of UVB irradiation. (**G**) Quantitative measurement of cell apoptosis under different doses of UVB irradiation. The apoptosis was detected by AO staining. One-way ANOVA test was conducted to determine the significance. Column with the same label indicates statistic insignificance. Semi-thin section was conducted to compare the morphology in caudal fin at different doses from 0 (**I**), 100 (**J**), 300 (**K**), and 500 (**L**) J/m^2^. The cellular organization of a cross-section of caudal fin is illustrated in (**H**). Scale bar = 25 um in (**I**–**L**).

Detection and activation of UVB inducible biomarkers were optimized using different non-lethal UVB doses. Zebrafish 3 dpf (days post-fertilization) embryos were irradiated at different doses of UVB from 100 to 1000 J/m^2^ (Figure 1A–E) and later fixed at 4 dpf to calculate the caudal fin size and relative apoptosis level. The results showed that the size of caudal fin was significantly reduced when irradiated with UVB higher than 300 J/m^2^ (Figure 1C–E and statistically compared to Figure 1F) compared to the control (Figure 1A). The surface area of the caudal fin decreased 40% (0.59 ± 0.05) for the group exposed to UVB at 300 J/m^2^, compared to the group without UVB treatment or with UVB exposure at 100 J/m^2^. Moreover, quantification of cells within the red square in caudal fin area (Figure 1A) that underwent apoptosis with acridine orange (AO) staining was captured under fluorescence microscopy. Apoptotic cell numbers also sharply increased in the groups exposed to UVB at the dosage equal to or higher than 300 J/m^2^ (Figure 1C–E and statistically compared in Figure 1G). Apoptosis was induced in 17% of cells in the surface area of caudal fin (17.05 ± 3.32) exposed to 300 J/m^2^ UVB, while cells exposed to 100 J/m^2^ UVB had no-to-little (1%) apoptosis. Under 1000 J/m^2^ UVB exposure, 17.48% (17.48 ± 8.21) fin area expressed apoptotic phenomenon, corresponding to fin shrinkage phenotype.

Semi-thin sections were used to evaluate the morphological changes of the caudal fin after UVB irradiation. Generally, during normal development, the skin of fin is divided into three layers. One to two layers of epithelial cells comprised the outer epidermis layer (purple-blue squamous cells). The inter-space between the epithelial cells is actinotrichia that produces collagens. The inner dermis cells (mesenchymal cells) lie under the epidermis stained a dark-blue color. The center part of the conjunctive tissue was stained a light purple (Figure 1H). Results showed the mesenchymal cells inside the caudal fin were very sensitive to UVB irradiation than other cellular compartments. Even in low dosages of 100 J/m^2^ UVB, while caudal fin size did not change and apoptosis was not yet activated, the mesenchymal cells were disorganized and missing (Figure 1J). When UVB dosage was higher than 300 J/m^2^, more apoptotic cells with a condensed nucleus were detected in epidermal layer (Figure 1K,L, highlighted by stars). Also, mesenchymal cells were more vulnerable than epithelial cells after exposure to UVB irradiation. Therefore, the optimized UVB dosage of 300 J/m^2^ will be used in the following experiments.

**Figure 2:**
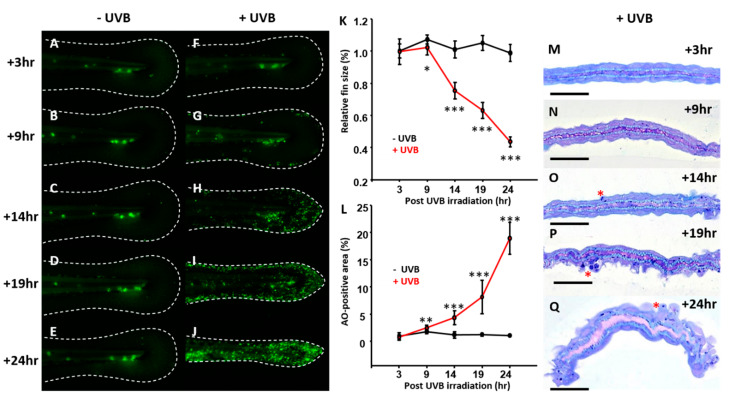
Time course changes of caudal fin in zebrafish after UVB irradiation. Zebrafish embryos aged 3 dpf were irradiated with UVB at doses of 300 J/m^2^. (**F**–**J**) The embryos were then collected at 3, 9, 14, 19, and 24 h after irradiation and subjected to AO staining to detect apoptosis and measuring the relative size of caudal fin. The control group was for comparison (**A**–**E**). (**K**) Dynamic changes of the relative caudal fin size in zebrafish with UVB irradiation at dose of 300 J/m^2^. (**L**) Dynamic changes of the relative apoptotic cells with UVB irradiation at a dose of 300 J/m^2^. (**M**–**Q**) Semi-thin sectioning was conducted to compare the interior morphology of caudal fin under UVB irradiation at a dose of 300 J/m^2^. (*n* = 5–15. Student’s *t*-test was performed on data from experiments. * *p* < 0.05, ** *p* < 0.01, *** *p* < 0.001). Scale bar = 25 μm in (**M**–**Q**).

Dynamic changes in the apoptotic events were monitored in order to understand time point when the apoptosis could be triggered after irradiation with the optimal UVB dosage of 300 J/m^2^. Zebrafish embryos aged at 3 dpf were irradiated with UVB at 300 J/m^2^ and later fixed at different time points to calculate the caudal fin size and relative apoptosis area. In terms of fin size, there was a significant reduction in the fin size from 9 h onwards post-irradiation (Figure 2F–J with statistical comparison in Figure 2K) when compared with the control group (Figure 2A–E). Consistent with the fin size reduction, by either AO staining (Figure 2G–J) or semi-thin section (Figure 2M–Q), a sharp increase in the apoptotic cells were observed from 9 h onwards post-irradiation at the dosage of 300 J/m^2^. According to these observations, the optimized UVB dosage at 300 J/m^2^ can be considered as a minimal effective dose and will be applied to all the following experiments.

### 3.2. DNA Damage Induction, ROS Release, and Inflammatory Response after UVB Irradiation

**Figure 3:**
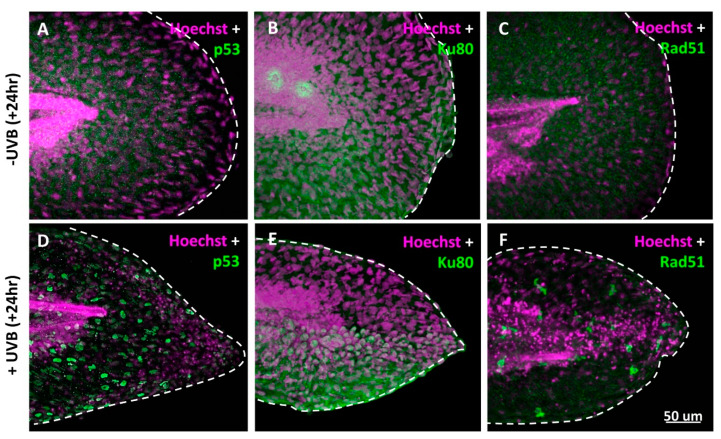
UVB activates p53 and DNA damage in zebrafish. Embryos aged 3 dpf were irradiated with UVB (+UVB) at a dose of 300 J/m^2^. Twenty-four hours later, the irradiated embryos were fixed and stained with p53, Ku80, and Rad51 antibodies. Embryos without (**A**–**C**) or with UVB irradiation (**D**–**F**) were stained with p53 (green) and Hoechst (purple) antibodies and detected under high-resolution confocal microscopy. The p53 protein translocated to the nucleus of cells undergoing apoptosis at 19 h after 300 J/m^2^ UVB irradiation.

In cell-based studies, UVB irradiation can trigger cell death, activate p53, release ROS, and induce inflammatory response. The researchers addressed the issue of similarity between zebrafish and human after UVB irradiation. Zebrafish embryos aged 3 dpf were irradiated with UVB at 300 J/m^2^ and later fixed at 4 dpf. Immunostaining with antibodies against p53, Rad51, and Ku80 was performed to detect DNA repair status. Tumor protein p53 is a key checkpoint protein on cell cycle and apoptosis in both zebrafish and humans [29]. After induction of UV-mediated DNA damage, activated p53 arrests the cell cycle to help cells repair DNA damage. Once the damage is too severe to restore, p53 then triggers cell apoptosis to prevent the oncogenic transformation [30]. Recombinase Rad51 and Ku80 are both DNA repair-related proteins that can be activated to repair the damage of double-stranded DNA by either non-homologous end-joining (NHEJ) or homologous recombination. Based on the facts, it was found that p53 (Figure 3D), Ku80 (Figure 3E), and Rad51 (Figure 3F) were indeed activated in the caudal fin area of zebrafish after UVB irradiation. In addition, we also noticed the activated p53 proteins translocated into cell nucleus of keratinocytes when embryos were irradiated by UVB (Figure 3D).

**Figure 4:**
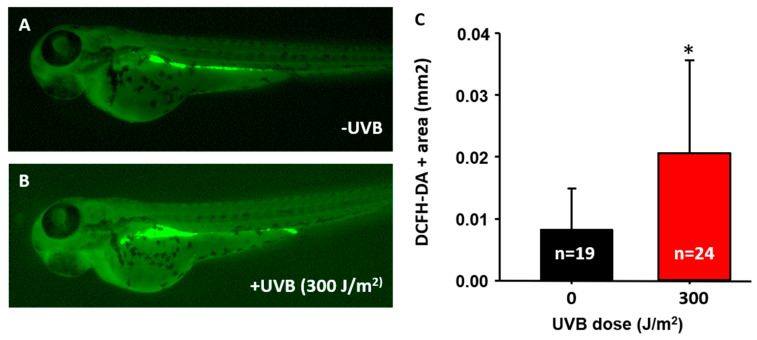
UVB induces zebrafish cells to produce reactive oxygen species (ROS). Embryos aged 3 dpf were irradiated with UVB at 300 J/m^2^. Embryos were stained with 40 μM DCFH-DA to detect the ROS. ROS expression without (**A**) and with (**B**) 300 J/m^2^ of UVB irradiation at 4 dpf. (**C**) The intracellular ROS signals were statistically quantified by the relative intensity of ROS fluorescent signals. ROS increased after exposure to UVB at 300 J/m^2^. (*n* = 19–24. Student’s *t*-test was performed on data from experiments. * *p* < 0.05).

In mammals, the UVB irradiation triggered the release of ROS to denature the lipid, nucleic acid, and protein [31]. To detect ROS production in zebrafish embryos, a ROS-sensitive dye of dichloro-dihydro-fluorescein diacetate (DCFH-DA) was used. Dichloro-dihydro-fluorescein diacetate is a cell-permeable dye that can release DCFH after hydrolysis by esterase. The colorless DCFH will be converted into 2’,7’-dichlorofluorescein (DCF) and simultaneously emit green fluorescence [32]. Zebrafish embryos at 3 dpf were challenged with UVB irradiation and later recovered for 24 h. By 4 dpf, embryos were incubated with 40 μM DCFH-DA to measure the level of ROS. The results showed that the relative area of DCFH-DA-induced fluorescence in the digestive track was significantly higher in the UVB-irradiated group (0.021 ± 0.015 mm^2^, *n* = 24, Figure 4B,C) than in the control (0.008 ± 0.006 mm^2^, *n* = 19, Figure 4A,C). This indicates that ROS was released in a UVB dose-dependent manner. It is also interesting to note that the gut tissues displayed very strong ROS-positive signals in those untreated control embryos (Figure 4A). The reason might be associated with the high affinity of DCFH-DA to gut lumen. A similar result has also been reported in a previous study done by Shi and colleagues et al. (2014) [33]. Although high in background nature, we were still able to do signal quantification and discovered the UVB-treated embryos, indeed, display more robust ROS signals (Figure 4B) than the control group (Figure 4A).

**Figure 5:**
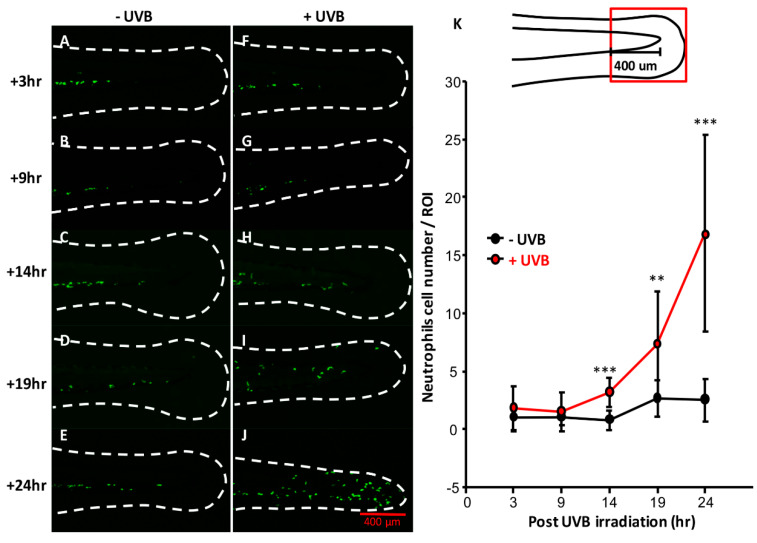
UVB irradiation induces inflammation in zebrafish. Neutrophils reporter line Tg (*mpo*: GFP) was generated to detect inflammation. (**A**–**E**) Embryos aged 3 dpf without UVB irradiation at different time points. (**F**–**J**) Neutrophils number of caudal fin area over the intermediate cell mass from 3 h to 14 h post-irradiation was counted. (**K**) Number of neutrophils within 400 μm region (red box) was calculated. (**F**–**G**) Neutrophils did not shift to caudal fin before 9 h post-irradiation. (**H**) After 14 h post-irradiation, neutrophils started to migrate to caudal fin. (**I**,**J**) Neutrophils continuously migrated from 19 h after irradiation to 24 h. (**K**) Quantification of the neutrophils at caudal fin. Neutrophils migration to caudal fin begins from 14 h post UVB irradiation and continued to 24 h post UVB irradiation. (*n* = 10–20, *t*-test was used to determine the significance change, * *p* < 0.05, ** *p* < 0.01, *** *p* < 0.001).

In mammals, a non-coding RNA released from the damaged skin cells trigger the TNF-α and IL-6 production to induce inflammatory response after UVB irradiation [34]. To monitor inflammatory response in vivo, transgenic Tg (MPO:GFP) zebrafish was used, which specifically expresses GFP in the neutrophilic lineage [35]. The Tg (MPO:GFP) transgenic zebrafish were irradiated with UVB at 300 J/m^2^ and the relative number of neutrophils in the caudal fin area was monitored. Generally, the neutrophils aggregated around intermediate cell mass and distributed less in the caudal fin area (Figure 5A–E). However, from 14 h onwards post-irradiation, the neutrophils gradually migrated to the caudal fin area that was exposed to UVB irradiation. We quantified the number of neutrophils within 400 μm length of fin area (red square). From 14 h onwards post-UVB irradiation (Figure 5H–K), the migrated neutrophils were significantly higher than those in control (Figure 5C–E,K).

### 3.3. Screening on Novel UVB-Inducible Markers by Microarray Analysis

**Figure 6:**
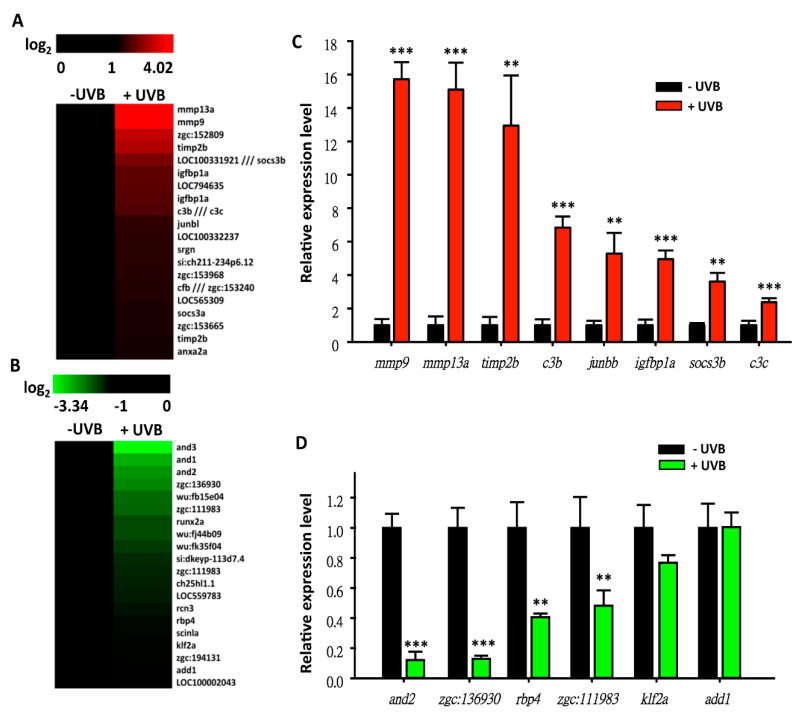
Microarray profiling of gene expression in zebrafish embryos after challenging with UVB irradiation. Heat map showed the upregulated genes (**A**) and the downregulated genes (**B**) with >1.5-fold difference after UVB irradiation. Validation of expression levels of upregulated (**C**) and downregulated (**D**) genes by qRT-PCR. These data showed log-ratios from a two-channel microarray. Gene expression of zebrafish aged 4 dpf was detected after UVB irradiation at 3 dpf. (Three replicates were performed in RT-PCR, and a *t*-test was used to determine the significant difference. * *p* < 0.05, ** *p* < 0.01, *** *p* < 0.001).

According to the above evidence, it was suggested that the basic cellular responses between human and zebrafish are similar, and that zebrafish may be a solid animal model to study the potential effects of UVB irradiation on gene expression profiling. In addition, previous studies showed that UV-induced the secretion of transforming growth factor (TGF)-β1 in fibroblast to activation protein (FAP)-α [36]. Also, one of the well-known inflammatory factors, nuclear factor κB (NF-κB), was activated and associated in neutrophils’ wound response in basal keratinocytes [37]. Moreover, a study confirmed that skin keratinocytes in zebrafish respond to the environment and trigger immune mechanisms when facing stress which is similar to humans [38]. Therefore, the UVB-induced zebrafish model in this study can be solid evidence to evaluate the UVB-associated pathway that may reflective of the human mechanism. To screen novel UVB-inducible molecular markers, zebrafish embryos aged at 3 dpf were irradiated with the optimized UVB dose (300 J/m^2^). By 4 dpf, total RNAs were extracted and subjected to Affymetrix (Santa Clara, CA, USA) GeneChip^®^ Zebrafish Genome microarray analysis. Two hundred and eighty-six probes (169 up, Table S2, and 117 down, Table S3) showed differential expression patterns between the UVB-irradiated and control groups (based on 1.5-folds cut-off threshold). After filtering the redundant and unannotated probes by GeneSpring software, it was noted that 248 genes (144 up- and 104 downregulated) showed significant differences. The top 20 ranking genes in the up- (Figure 6A) and downregulated (Figure 6B) groups are represented as heat maps to show their relative expression level. Among these genes, *mmp13a* and *mmp9* were the most highly upregulated and *and1-3* were the most downregulated. To validate the array data, eight upregulated genes (*mmp9, mmp13a, timp2b, c3b, junbb, igfbp1a, socs3b*, and *c3c*) and six downregulated genes (*and2, zgc: 136930, rbp4, zgc: 111983, klf2a*, and *add1*) were selected and subjected to quantitative real-time PCR (qRT-PCR) to measure the gene expression level (Figure 6C,D). For example, the highly upregulated targets in microarray, *mmp9* and *mmp13a*, showed 15.7- and 15.1-fold induction by qRT-PCR, indicating that zebrafish microarray analysis is plausible and reasonable (Figure 6A,B).

**Figure 7:**
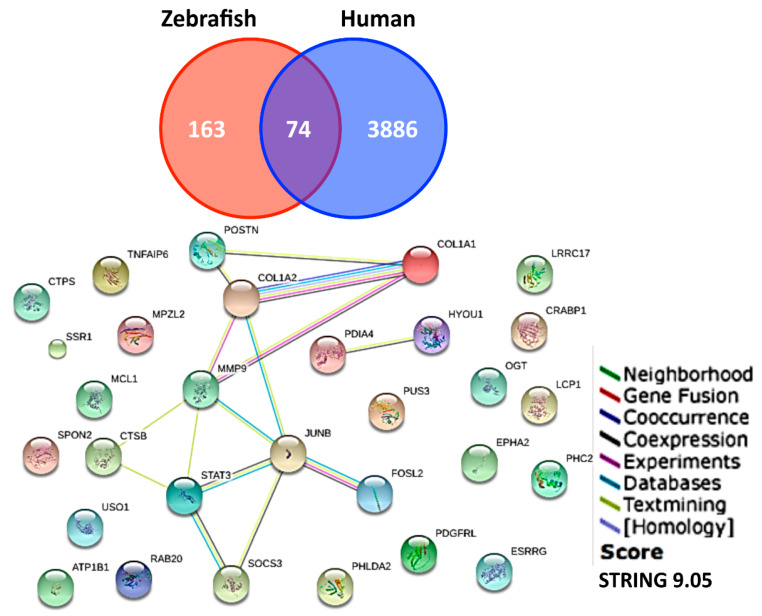
Functionally overlapping genes were compared between humans and zebrafish. (**Upper panel**) 74 genes’ expression were conserved between zebrafish and human skin (from literature) after UVB irradiation according to microarray data. (**Lower panel**) The overlapping expression genes were analyzed by STRING, a known database which predicts protein interactions including direct (physical) and indirect (functional) associations. The functions of overlapping genes between human and zebrafish are related to tissue reconstruction.

Among these 248 genes, gene ontology was performed using DAVID for gene enrichment analysis. Different gene expressions were enriched in clusters of cytoskeletons (enrichment score 4.1), proteolysis (enrichment score 2.38), extracellular region part (enrichment score 2.22) and other pathways (Figure 7). For example, the Extracellular Matrix (ECM) genes like *collagen* and *fibronectin*, were downregulated, whereas the proteolytic genes, *mmp13a* and *mmp9*, were upregulated. Two hundred and forty-eight differential expression genes were subjected to Search Tool for the Retrieval of Interacting Genes/Proteins (STRING) to analyze the potential protein–protein interaction networks.

A cross-species chip-to-chip comparison was performed to explore the gene ontology and analyze gene clusters in zebrafish and humans [20] after UVB irradiation. With a cut-off of 1.5-fold differential expression level, genes were listed common to both zebrafish and humans (Table S4). With pathway analysis, we identified these key hub genes that might play important roles during UVB-irradiation response (Figure 7).

### 3.4. Temporal and Spatial Expression Patterns of UVB-Inducible Markers

**Figure 8:**
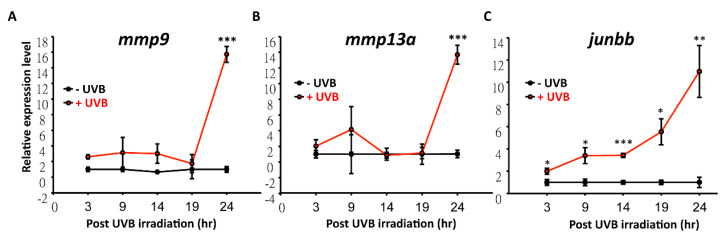
The temporal expression profiling of *mmp9*, *mmp13a* and *junbb* after UVB irradiation in zebrafish by quantitative real-time RT-PCR. (**A**) *mmp9* and (**B**) *mmp13a* were not activated until 24 h after UVB irradiation. (C) *junbb* was sharply activated from 3 h onwards after UVB irradiation. The relative expression level for each transcript was normalized with *β-actin*. (Three replicates were performed in RT-PCR and the *t*-test was used to determine the significance of the results. * *p* < 0.05, ** *p* < 0.01, *** *p* < 0.001).

According to Database for Annotation, Visualization, and Integrated Discovery (DAVID) analysis, it was found that *mmp9* and *mmp13a* showed multi-functions during UVB irradiation (Figure 7 and Table S4). Expression level of *mmp9* and *mmp13a* under serial dosages of UVB irradiation were detected. There was no obvious difference between 0 to 200 J/m^2^. However, expression of *mmp13a* and *mmp9* surged to 16- and 13-fold, respectively, after challenging with 300 J/m^2^ of UVB irradiation (Figure 8A,B). In addition, expression levels of *mmp9* and *mmp13a* within 24 h post-irradiation was traced and surged at this time point. Surprisingly, one of the genes that was identified from DAVID, *junbb*, significantly increased to 3.5- and 5.5-folds within 9 h and 19 h (Figure 8C). This suggests that *junbb,* instead of *mmps,* may serve as a novel and early onset biomarker for UVB-mediated response, not only in zebrafish, but also in humans. In addition, genes can be further categorized into two sub-groups including early and late UVB-inducible markers by systematic analysis. For early markers like *junbb*, its relative expression was already elevated as early as 3 h post UVB irradiation and showed continuous elevation over time. For late markers like *mmp9* and *mmp13a*, its relative expression did not increase until 24 h post-UVB irradiation.

## 4. Discussion

**Figure 9:**
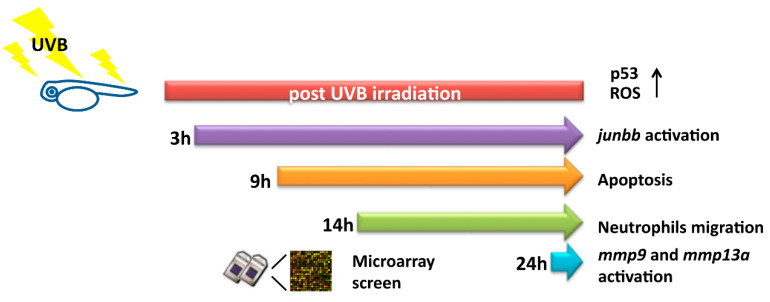
Summary of the cellular responses and gene expressions after UVB irradiation in zebrafish. *Junbb*, *mmp9,* and *mmp13a* were identified as UVB-inducible markers by using microarray screening.

In previous studies, the biological effect after UV exposure was validated through myriad models, such as in vitro skin reconstitution [21,39], human keratinocytes [40,41], and transgenic mouse [42,43,44], for specific UV-related research. Although in vitro model shows great convenience and provides rapid screening on UV-induced phenotypes, a lack of systematic interaction in whole skin structure to mimic real UV response is always a barrier that researchers need to overcome. Therefore, a zebrafish model and whole organism gene profiling approach was adopted and leads to identification of a novel biomarker of *junbb* showing more sensitivity than *mmps* which has been used in mouse model (summarized in Figure 9).

Several parameters have been used to evaluate responses to UVB, such as ROS generation [45], number of UV-induced apoptotic cells, and caudal fin measurement [46]. In this study, quantification of caudal fin shrinkage and apoptotic area to validate the degree of UVB damage was established. The results showed that zebrafish showed a decrease in the size of caudal fin and an increase in apoptotic cells after 300 J/m^2^ UVB exposure. Any dosage higher than 300 J/m^2^ could induce UVB responses; however, both fin shrinkage and number of apoptotic cells both reached to a plateau that showed no significant induction with exposure over 300 J/m^2^. In addition, the structure of caudal fin was severely deformed at the dosage of 300 J/m^2^. Another study by Tsai and colleagues [47] demonstrated the flavone effect on UVB-exposed zebrafish. In their study, 100 mJ/cm^2^ (equal to 1000 J/m^2^) of UVB irradiation was subjected to zebrafish aged at 3 dpf to induce fin damage that was recovered by flavone treatment. The dosage of UVB irradiation was from 3 to 120 mJ/cm^2^ and the NF-kB pathway was effectively and significantly induced under 24 to 48 J/cm^2^. In Tsai et al.’s (2012) [47] study, the morphological changes were not addressed and the inflammatory response for several cytokines were not activated until 15 to 20 h post-irradiation. In another study, a high dosage of UVB irradiation (1.08 KJ/m^2^) during embryonic development was given to induce p53-associated DNA repair pathway [48]. Compared to the above previous studies, we optimized the UVB irradiation dose at 300 J/m^2^ which was the minimum effective dosage (MED) to induce UVB corresponding effects on morphological, cellular, and molecular levels in zebrafish.

Recently, one of the CNC-bZIP transcription factor family members, Nfe2-related factor (Nrf)-3, was suggested for its role during UV-induced keratinocyte apoptosis in mice and humans [49], which was considered to have a response in cellular redox status in zebrafish [50]. The similarity shared between mammals and fish suggested to us that the pathway in response to UV radiation may be shown to be in common. It has been well-known that p53 is involved in DNA damage after UVB irradiation [30,51]. Similar to mammals, UV-induced DNA damage activates the p53 pathway to delay the cell cycle, repair damaged nucleotides, or turn on the apoptotic mechanism in zebrafish model [52]. In this study, p53 activation (by whole-mount immunostaining) at 24 h after 300 J/m^2^ UVB exposure in UVB-induced zebrafish model was demonstrated. Not only p53, but also Ku80 and Rad51 that is involved in NHEJ (non-homologous end joining) and homologous recombination were activated in the said model system. Considering the benefits of easy observation and maintenance, zebrafish serves as a great model to study UV-associated pathways that can stimulate comparable factors in mammals. In human and mouse, *mmp1, mmp2, mmp3*, and *mmp9* were highly expressed after exposure to UV irradiation [53,54,55]. Consistent with our microarray profiling, *mmp13a* and *mmp9* were the most highly activated genes when zebrafish were treated with 300 J/m^2^ UVB irradiation. It suggested that *mmp*-associated effects, such as inflammatory reaction [56], tumor invasion [57], and cutaneous aging [58] are conserved between zebrafish and humans. In addition, a significant number of neutrophils were increased within caudal fin area, which inferred the stimulation of inflammatory response after UVB induction. However, the relative late induction of *mmps* draws our concern on looking for other novel candidates during UVB irradiation. The quick and sharp response of novel UVB-associated marker, *junbb*, was identified and validated in this study. *Junbb* has been reported previous by Ishida and colleagues [59] as a regeneration biomarker after tail fin amputation in zebrafish. While *Junbb* belongs to the JunB proto-oncogene (AP-1 transcription factor subunit), it plays a role as a potent transcription factor on mediating cell proliferation [60,61].

## 5. Conclusions

In this study, *junbb* was discovered to significantly respond to UVB as early as 3 h post-irradiation which may be a permissive marker for monitoring if cells are undergoing UVB-induced stress and cell damage. In the future, by generating a transgenic line carrying a *junbb* reporter, we might be able to build up a useful tool to monitor UVB-induced damage in real-time and use this platform to screen drugs or small molecules that can protect cells from UV damage. To sum up, this study has indeed showed improvement of the model system and novelty on gene expression analysis after UVB exposure. The early onset of *junbb* provide us a UV-induced marker that has a great potential to serve as a quick and accurate technique in accelerating drug screening and development in the pharmaceutical field.

## Supplementary Materials

The following are available online at https://www.mdpi.com/2076-2615/10/6/1096/s1, Table S1. Primer information used to perform real time RT-PCR. Table S2. The 1.5-fold upregulated gene list after exposed to UVB irradiation at 300 J/m^2^. Table S3. The 1.5-fold downregulated gene list after exposed to UVB irradiation at 300 J/m^2^. Table S4. The up- and down-regulated gene list that can be functional grouping by DAVID annotation.
